# Behavioural plasticity in activity and sexual interactions in a social lizard at high environmental temperatures

**DOI:** 10.1371/journal.pone.0285656

**Published:** 2023-07-26

**Authors:** Nicola Rossi, Margarita Chiaraviglio, Gabriela Cardozo

**Affiliations:** 1 Universidad Nacional de Córdoba, Facultad de Ciencias Exactas Físicas y Naturales, Laboratorio de Biología del Comportamiento, Córdoba, Argentina; 2 Consejo Nacional de Investigaciones Científicas y Técnicas (CONICET), Instituto de Diversidad y Ecología Animal (IDEA), Córdoba, Argentina; University of Regina, CANADA

## Abstract

Sexual selection often shapes social behavioural activities, such as movement in the environment to find possible partners, performance of displays to signal dominance and courtship behaviours. Such activities may be negatively influenced by increasing temperatures, especially in ectotherms, because individuals either have to withstand the unfavourable condition or are forced to allocate more time to thermoregulation by increasing shelter seeking behaviour. Thus, they “miss” opportunities for social and reproductive interactions. Moreover, behavioural displays of ectotherms closely depend on temperature; consequently, mate choice behaviours may be disrupted, ultimately modifying sexual selection patterns. Therefore, it would be interesting to elucidate how increasing temperatures associated with global warming may influence activity and social interactions in the species’ natural habitat and, specifically how high temperatures may modify intersexual interactions. Consequently, our aim was to explore differences in the daily pattern of social interactions in an ectotherm model, *Tropidurus spinulosus*, in two thermally different habitats and to determine how high temperatures modify mate choice. High environmental temperatures were found to be associated with a bimodal pattern in daily activity, which was closely linked to the daily variations in the thermal quality of the habitat; whereas the pattern and frequency of social displays showed less plasticity. The time allocated to mate choice generally decreased with increasing temperature since individuals increased the use of thermal refuges; this result supports the hypothesis of “missed opportunities”. Moreover, at high temperatures, both sexes showed changes in mate selection dynamics, with females possibly “rushing” mate choice and males showing an increase in intermale variability of reproductive displays. In our ectotherm model, plastic adjustments in the behavioural activity pattern induced by high temperatures, plus the modification of the displays during courtship may ultimately modify mate choice patterns and sexual selection dynamics.

## 1. Introduction

The behaviour of ectotherms has been the focus of research in recent decades, since it plays an important role in several aspects of their biology [1]. However, since behaviour is a temperature-related trait, it is under increasing pressure due to global warming [2, 3] and ectotherms are among the groups most susceptible to high temperatures [4]. Many ectotherms carefully regulate their activity time to adjust their daily needs (e.g. foraging, thermoregulation, habitat defence, social interactions) to suitable environmental temperatures; thus, they avoid overheating, which can trigger a heat stress response [4, 5]. Moreover, low plasticity in upper critical temperatures suggests a limitation of ectotherms to adapt to warming environments [6]. High temperatures increment the kinetic energy of biochemical reactions and lead to an increase of the metabolic rate [6, 7]. This metabolic effect may extend to a higher level of body organization, ultimately influencing the decision-making process of the individual [1]. Indeed, high temperature may induce changes in behaviour, for example in the pattern of movement related to shelter seeking in both thermoconformers and thermoregulators [8]. However, how behavioural changes modify social interactions, particularly reproductive ones, has been poorly studied so far in the light of global warming [9–11]. Therefore, an interesting question is how increasing temperatures may influence both the daily pattern of social interactions of ectotherms and intersexual interactions.

Social interactions during the reproductive season are often modelled by sexual selection, i.e. a powerful evolutionary force that may favour or hinder the adaptation of a species to environmental stressors [12]. Pre-copulatory sexual selection involves several social activities that may contribute to an increase of individuals’ fitness, such as behavioural displays to signal their dominance and settle territorial boundaries [13], movements to find possible partners [14] and courtship behaviours [15]. However, such activities may be negatively influenced by increasing temperatures, because individuals either have to withstand the unfavourable condition by taking metabolic risks, or are forced to accommodate their time budgets to thermoregulate at the expense of other activities, such as intraspecific interactions [16–19].

It has been observed that a thermally stressed state makes lizards initiate a shelter-seeking behaviour [8]. Plasticity in behavioural thermoregulation, particularly shifts in microhabitat use like retreating into refuges, is an effective way to maintain their body temperature within their preferred range [6, 20, 21]. However, according to the “missed opportunities” hypothesis [22], when thermal quality of the environment (i.e the difference between the mean hourly operative temperature, T_e_, in the habitat and the set-point range of the species) is poor due to high temperatures, individuals need to spend more time shuttling between sun and refuges [8, 23]. Thus, the trade-off between searching for preferred microclimates and performing other activities, such as social and reproductive ones, tilts towards the former [24, 25]. One useful approach to the study of the plastic capacity of a species to thermoregulate in its natural habitat at increasing temperatures is the space-for-time substitution design [26], in which divergences in climatic factors, such as temperature, between the habitats of spatially separated populations of a single species are taken as an estimation of projected temperature increments. This experimental design has been used to study phenotypic plasticity in ectotherm models [3, 27, 28].

Behavioural plasticity also plays a central role in mate choice, since displays may be used as a signal for the other sex [29]. Several ectotherm species show evidence of both female and male mate choice [30–32], with individuals of both sexes being often selected for characters that are related to their reproductive potential [31, 33–35].

Mate choice is often costly because individuals must devote time and energy to find a mate and perform behavioural displays, and environmental factors can increase such costs [36]. Suitable environmental temperatures allow individuals to allocate time to mate search, courtship and selection [37, 38]. However, individuals that engage in courtship or reproductive behaviours at high temperatures are at increased risk of overheating and may seek thermal refuge, thus losing mating opportunities. They may also not be able to select a partner, since the production of behavioural displays closely depends on temperature [39]. In endotherms such as zebra finches, females select males randomly at high temperatures, possibly due to the effect of heat stress on cognitive capacity, or to the distraction caused by heat, i.e. females must divert their attention to the heat stressor, paying less attention to the mate selection process [40]. Likewise, in the jumping spider *Habronattus clypeatus*, high temperatures affect the courtship displays of males by modifying the frequency and speed of vibratory displays. However, female selection may not match such changes in male displays, therefore reproductive male signals are decoupled from female preference [41].

Lizards are an interesting model to study social interactions in warming environments [42] because they are ectotherms and exhibit a marked social behaviour and interesting sexual selection dynamics [43, 44]. Males may move between perch positions to attract females and repel other males [45–47], and may engage in behavioural displays to communicate with conspecifics [48–50]. Female and male mate choice has been reported in lizards and is often based on visual phenotypic traits [51–54]. In particular, it has been long known that colouration is an important signal for lizards in sexual selection contexts. Males may express their quality by varying colouration intensity, hue or extension. For example in the Agamidae family male lateral colouration is under sexual selection [55–57]. Indeed male Indian rock agamas show strikingly different colours during courtship displays to entice a possible partner [58]. On the other hand, females may be selected for their body size, which is correlated with high fecundity in many species [59]. Moreover, phenotypic variation in female lizard is related to diversity in reproductive strategies [60]. However, selection patterns of both sexes might vary because mate choice is context-dependent and might be affected by the thermal quality of the environment [4]. Behavioural displays are energetically costly for lizards; indeed, to perform a short but intense bout of physical exercise, males may rely heavily on anaerobic metabolism, which can impair lizard movements and make them more vulnerable [61]. High temperatures may exacerbate such costs [46, 62], even leading to a loss of mating opportunities, because lizards will have to reroute from courtship displays to behaviours that avoid overheating [63, 64]. Furthermore, male behavioural displays have different energetic costs [65]; for example, Headbobs are often considered “cheaper” than Pushups [61]; therefore, lizards may choose to alter the frequency or the type of behavioural displays to elicit different responses in females [66]. However, the effect of high temperatures on the choice of displays to interact between sexes has still not been studied.

In this study, we combined two complementary approaches in the model lizard species *s*: a space-for-time substitution field design, which was used to study variations of the daily activity pattern of social interactions according to temperature variation in the species natural habitat, and an experimental laboratory design, in which the thermal environment was finely controlled to specifically assess variations in intersexual interactions. We hypothesize that increasing temperatures may impact T. spinulosus behaviour, forcing individuals to plastically modify their behavioural activity pattern. This modification would affect the performance of displays and the time allocated to social interplay; thus, individuals would mis opportunities for sexual interaction. Consequently, for the field design we predict a variation in the daily activity pattern of lizards between habitats differing in thermal profiles and according to the operative temperatures that are within the preferred range of the species. Moreover, since to avoid overheating individuals seek refuge more often, we also predict that at high temperatures, lizards will allocate less time to perching in simultaneous presence with conspecifics, to performing behavioural displays, and to moving between perch positions. Likewise, we expect high temperatures to affect mate choice because the rate of male Pushups in favour of Headbobs and the time focal individuals engage in interactions with the possible partners are reduced, leading to increasingly random patterns of selection.

## 2. Materials and methods

Study species: *Tropidurus spinulosus* is a member of the Tropiduridae family, which includes many social lizard species [67]. In particular, in *T*. *spinulosus* individuals are often observed in groups on rocky outcrops, interacting with one another [49]. It shows a narrow range of preferred temperatures: selected temperature (Tsel) = 33.7°C, first quartile of the preferred range (Tset-min) = 32.2 and third quartile of the preferred range (Tset-max) = 35.2 [68]. Its reproductive behaviour involves several displays, like Pushups and Headbobs, and active selection of mates [69, 70]. Since female body size is correlated with fecundity, it may be an object of selection by males [71]. On the other hand, male-biased dichromatism in body parts linked to conspecific interactions [49] can be correlated with reproductive potential, e.g., male flank colouration is correlated with testicular volume, being possibly involved in mate selection [72]. Moreover, our study population lies at the southernmost and coldest point of the species and family distribution, where global warming may have a notable effect [73]. All these facts support *T*. *spinulosus* as a suitable model to study the effect of temperature on behaviours associated with sexual selection dynamics.

## 2.1 Field study: Activity pattern and social interactions

### 2.1.1 Study site and design

We used a space-for-time substitution design, which consists of studying a species in habitats that differ in environmental factors, such as temperature, to a degree that simulates a forecast of global warming [26]. IPCC predicts a climate warming within the range of 2°C to 4°C by the end of the century [74, 75]; however, given the lack of short-term mitigation measures to reduce emissions, the likelihood of reaching a 4°C warming, as predicted by the Representative Concentration Pathway 8.5 (hereafter, RCP), has increased. We sampled a population of *T*. *spinulosus* that is distributed in two geographically adjacent but thermally distinct habitats in “Los Chorrillos Natural Reserve” (-31.390728°,-64.624645°; -31.405057°, -64.587391°): High Temperature Habitat (HIGH), where the rocky outcrops are completely exposed to sun radiation, and a Low Temperature Habitat (LOW), where the rocky outcrops are partially protected from the sun by shades cast by elements of the surrounding arboreal vegetation, thus generating patches of shade and patches of sunlight on the outcrop. The habitats are located in the same hilly region in Córdoba province, Argentina, and at an elevation of 900 m. For each of the two habitats, four rocky outcrops were selected in which 2 to 4 individuals were observed in an exploratory sampling. The average distance between the sampled rocks of the two thermal treatments was approximately 600 meters. Lizards occurring in the two thermal habitats show the same preferred temperature (T_sel_ HIGH: 33.94 ± 1.95; T_sel_ LOW: 33.30 ± 2.10; P-value = 0.10); therefore, any possible acclimation effects should be discarded (López Juri, unpublished data).

#### 2.1.2 Characterization of thermal habitat

To characterize the thermal environment, we recorded substrate temperature (Ts) in HIGH and LOW habitats using dataloggers (Model Ibutton DS1921G, Maxim Integrated). Dataloggers recorded instantaneous temperature at 5-minute intervals during the entire study period. Initially, in each rocky outcrop, two points where the angle of sun incidence varied were sampled: i) the highest perch position, and ii) a medium height perch position. However, temperature values of both perches were averaged because Ts did not differ significantly between positions (Student’s T = 1.40, P-value = 0.16). The mean difference in Ts between the HIGH and LOW habitats was 3.7±2.54°C, which closely matches the expected increase in surface temperature forecast by the extreme warming scenario of the IPCC (RCP 8.5).

Ts was also taken as a reliable estimation of the species operative temperature (Te), since the thermal biology of small reptiles (<50 g) is roughly estimated by a “naked datalogger” [76]. We then calculated the thermal quality of the habitats using the *de* index [77], i.e. the difference between the mean hourly Te and the Tset range [4, 78]. A habitat is considered of high thermal quality when Te is within Tset (*d*_*e*_ index = 0).

#### 2.1.3 Activity recordings

To characterize the activity pattern in both HIGH and LOW thermal habitats, we used camera trap (Bushnell, model Aggressor) recordings, for 20 days in October 2018, which includes the central period of the breeding season [68]. Camera traps were pointed at the perches used by lizards, as determined in previous exploratory studies of the rocky outcrops. We mounted the cameras on posts 5 meters away from the rocks to avoid disturbing animal activity. Such distance allowed us to scan an area of approximately 37.5 m^2^, which is similar to the home range area of other *Tropidurus* species during the reproductive season (in *Tropidurus itambere* 12.30 ± 17.78 m^2^ for females and 47.27 ± 56.75 m^2^ for males, [79]; in *Tropidurus torquatus* 37.0 ± 13.7 m^2^ for females and 50.8 ± 20.7 m^2^ for males [80]). A recording schedule was set for the cameras, which automatically triggered 1 minute of video recording with 5-minute intervals, from 0700 to 1900 hours. Thus, lizards were video-recorded for an average of 120 minutes during the diurnal phase of the natural photoperiod. To standardize weather conditions, cameras were operated on sunny days only [81].

#### 2.1.4 Video analysis

We obtained 7280 and 8320 minutes of video recording with presence of individuals for HIGH and LOW temperature habitats, respectively. Behavioural activity was scored from the videos using the software Solomon Coder, which allows us to manually record behaviours at each frame of the video [82]. We recorded the following variables:

- Active time (AT): time (seconds) spent by the individual outside the refuges, either moving or not [81].
- Simultaneous presence (SP): time (seconds) during which two or more individuals were active in the area captured by the camera trap; this variable was used to measure the time individuals dedicate to social activity.
- Pushup time (PT): time (seconds) during which males performed Pushups on the rock surface.
- Movement time (MT): time (seconds) spent by an individual in locomotion on the rock surface.

### 2.2 Laboratory experimental study: Mate choice

We performed mate choice experiments in a laboratory set up at different temperatures (see section 2.2.3) to assess whether the time spent near the potential partners as well as the selection outcome varied between thermal treatments. Focal individuals were exposed to two conspecifics of the opposite sex that differed in phenotypic traits related to the reproductive potential. For male mate choice trials, we considered female body size as selection character because snout-vent length (SVL) correlates with fecundity in terms of clutch size [71, 72]. For female choice trials, we considered male flank colouration as selection character, since it correlates with male reproductive potential in terms of testicular volume [72] (See section 2.2.3 for more details).

#### 2.2.1 Lizard capture and handling

To perform mate choice tests, lizards (N = 35 females; N = 42 males) were captured from Los Chorrillos private Natural Reserve during two consecutive breeding seasons (September to December, 2017–2018). Geographical coordinates of each collection site were recorded to release the lizards at the same site after the end of the experiments. In the laboratory, lizards were kept individually in plastic containers (30x25x20cm) enriched with rocks taken from the natural reserve and exposed to light, simulating a natural photoperiod (0900–1700 hours, Zoomed UVB 5.0 UV tubes) and air temperature of 28°C (the mean environmental temperature of the capture site) for 2–3 days until mate choice trials were performed. One larva of *Zophoba morio* and one of *Tenebrio molitor* were provided daily, and water was provided *ad libitum*.

#### 2.2.2 Phenotypic and reproductive characterization

Reproductive condition was evaluated by ultrasound scanning (Sonosite 180 Plus, transducer 5–10 MHz). We used receptive females with vitellogenic follicles and reproductive males with enlarged testes and sperm presence [83].

We measured SVL of both males and females using a digital calliper to the nearest 1 mm. Male colouration was characterized by taking pictures of lateral surfaces of the lizards. In previous studies, we characterized the dichromatic colours of the species from a lizard visual model using spectrophotometric data [49]. In the flanks, the dichromatism was mainly due to male-specific light blue spectra reflected at the bottom scales together with medium-wavelength green and long-wavelength yellow spectra. Thus, we quantified the area of blue, green and yellow bands by performing a supervised classification on pictures of the males’ flanks. Pixels sampled in 10 randomly selected individuals of each sex, which presented the dichromatic clusters mentioned above (Rossi et al., 2019), were used to produce training matrices corresponding to the three colour bands (the number of pixels sampled was related to the variability of each colour: yellow: 691 pixels, green: 1163 pixels, blue: 1836 pixels). Such matrices were used to assign each pixel of the flank of the males involved in the present study to a colour band through a supervised classification using the RandomForest function from the homonymous R package [84]. The algorithm for our dataset produced an out-of-bag error of 9.02%, which is considered acceptable [85, 86]. Green, blue and yellow relative proportions were summed, since together these colours of *T*. *spinulosus* flank cause the highest stimulation according to a lizard visual system [49]. However, using a logistic regression, we also tested which of the colour bands better predicts female choice. Since yellow was the best predictor (LR Chisq = 6.30 P = 0.01), we used it to compare female choice among thermal treatments.

#### 2.2.3 Design of mate choice trials

All mate choice experiments were conducted in a laboratory at three different temperature treatments: a current scenario and two warming treatments, according to the prediction of the RCP [74] for the end of the century. The current scenario was set at 34°C, which corresponds to the T_sel_ of the species [68]. The warming treatment set at 36°C simulated an intermediate rise in temperature (+2°C, RCP 4.5) and the 38°C treatment simulated an extreme increase of temperature (+4°C, RCP 8.5). Both warming scenarios are within the range of the temperatures selected by the species [68].

For the male mate choice trials, one focal male was presented with a dyad of females of different body size as a stimulus (at least 1 cm difference in SVL). For the female mate choice experiment, each focal female was presented with a dyad of males as a stimulus; males had a similar SVL (<0.3 cm of difference) but at least 20% discrepancy in area of flank colouration (sum of green, blue and yellow proportions). The total number of trials for male mate choice was n = 14 for each of the three temperature treatments (34°C, 36°C and 38°C), whereas for female mate choice, the number of trials was n = 11 for 34°C and n = 12 for 36°C and 38°C treatments.

Individuals participated as focal only for one trial, whereas dyad individuals were used for the three trials at different temperature treatments. All lizards were allowed to rest for one day between trials. After the experiment, lizards were checked for normal behaviour during one week [87] and then released at the capture sites.

**Fig 1:**
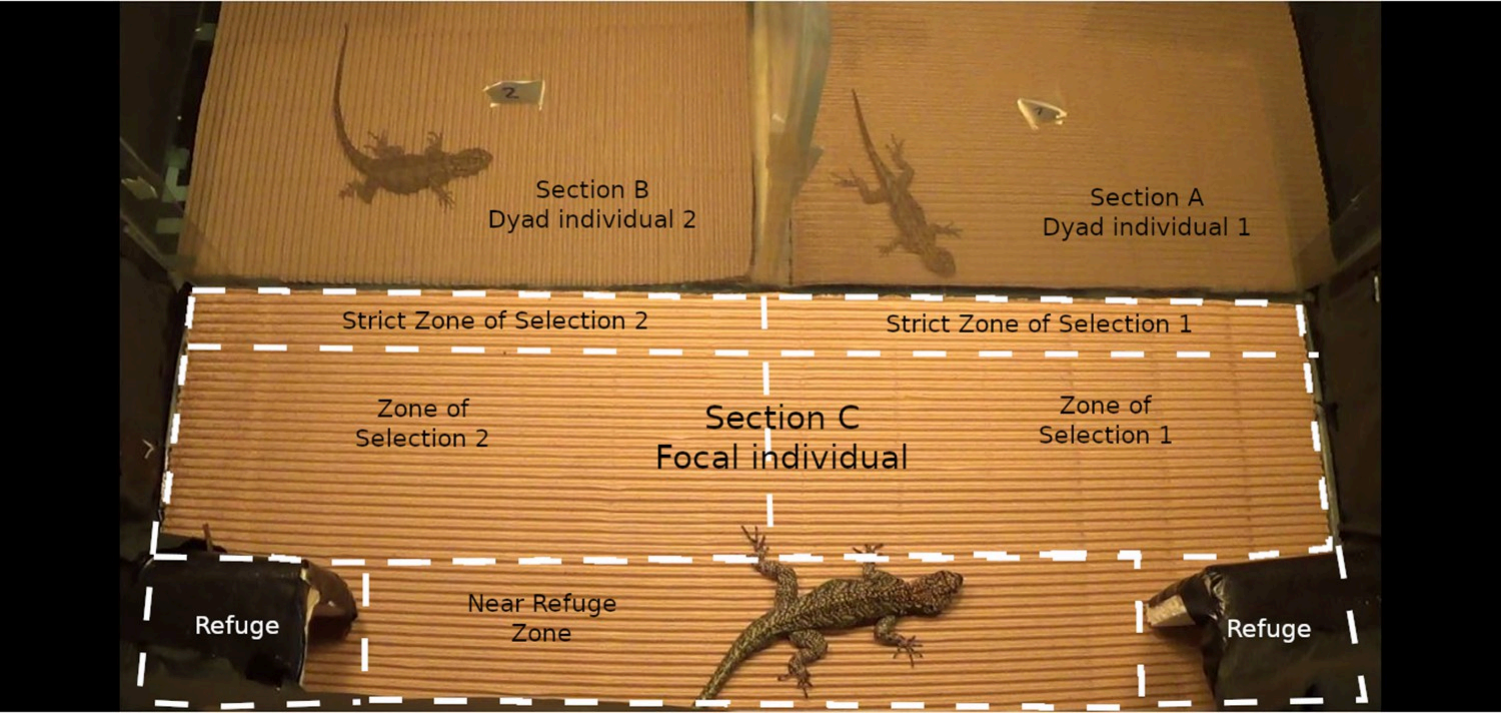
Arena for mate choice trials. White dashed lines delimit the apparatus in Any-Maze.

A three-section arena was used to conduct the trials (Fig 1). The two individuals of the dyad were placed in sections A and B, which were delimited with an opaque plastic divider to avoid any visual or physical contact. The focal individual was placed in section C, which was separated from the other sections by a transparent glass barrier, allowing visual but not physical contact with the dyad. Two Styrofoam^TM^ thermal refuges were placed in the corners of the arena in section C (7°C below the treatment temperature). The walls of the arena were covered with a black plastic film to avoid external visual interferences and the bottom was covered with light-brown corrugated cardboard to facilitate lizard movements. Three lamps (Osram 1500W) were suspended above section C so that treatment temperature had effect only on the focal section without significantly heating the dyad sections (difference <1° between the most extreme treatment and the current scenario in the dyad sections). The output of the lamps was adjusted with dimmers. Before the start of each experiment, temperature was measured at different points of section C to confirm thermal homogeneity (difference <0.5°C). At the beginning of the experiment, the focal lizard was placed behind a transparent plastic barrier in section C, which allowed visual evaluation of the dyad for 5 minutes; then, the barrier was lifted. Lizards were then recorded for 40 minutes. Afterwards, the experimental arena was cleaned with alcohol to eliminate any chemical trace of the individuals and the corrugated cardboard was replaced.

#### 2.2.4 Video analysis

The videos of mate choice trials were processed using ANY-maze (Stoelting.co, USA). For each video, an apparatus map was drawn on focal section C to define the following zones (Fig 1): i) Refuges; ii) Near Refuge Zone; the closest zone to both refuges where preference for a dyad individual cannot be established; iii) Zone of Selection 1 and iv) Zone of Selection 2; both indicate broad preference, since they are the proximal zones to dyad individuals 1 and 2, respectively; and v) Strict Zone of Selection 1 and vi) Strict Zone of Selection 2: the zones where the focal lizard touches the glass close to one of the dyad members, indicating an unequivocal interaction towards such individual.

In each potential mate choice trial, we recorded the following variables in the focal individuals [88–91]:

- R: Time spent in the Refuges to test the “missed opportunities” hypothesis.
- ZS: Time spent in the Zones of Selection.
- SZS: Time spent in the Strict Zones of Selection.
- Latency: measured as the time before an individual has access to a Zone of Selection.
- First access: the first Zone of Selection where the focal individual enters to evaluate the dyad individuals; it is often considered an expression of preference.
- Movement: Time spent moving in the arena, to test whether temperature influences the locomotory behaviour involved in mate selection.
- Headbobs: Number of Headbobs.
- Pushups: Time males spent performing Pushups.

### 2.3. Data analysis

In the field study, for each variable, we calculated the daily patterns in the two habitats by fitting a kernel probability function that summarized the density of observations during the sampled hours. We then calculated an index of overlap between HIGH and LOW habitat distributions by applying a Δ4 estimator [92], which calculates the area under the density distribution curves that is shared between habitats. The mean and confidence interval of the overlap estimator were calculated by bootstrapping the data [92]. Significant differences in daily patterns were assessed through a Watson’s large sample non parametric test [93]. We studied hourly differences in behavioural activities between habitats by applying a Chi-square test on the aggregated time data across rocks and days. Finally, we tested for differences in the total daily time allocated to a variable by modelling the activity data from each day of recording to a zero-inflated linear mixed distribution (*lme4* package, [94] with “Habitat” as a fixed factor and “Rock” as a random factor to account for data recorded in the same rocky outcrop.

For mate choice trials, we analysed female focal and male focal datasets separately. We analysed latency and Pushups performance through ANOVAs, with “Temperature” as fixed factor. Standardized residual plots were visually inspected to verify homoscedasticity; normality was assessed through a Shapiro-Wilk test on the residuals of the model. On the other hand, the time spent in NRZ showed strong deviations from normality; therefore we applied a non-parametric Kruskal Wallis test. For female time preference, we performed two analyses taking into account 1) the summed proportions of the colour bands of males and 2) only the yellow colour band, since it is the best predictor of female choice (see Section 2.2.2). Preference time was tested by applying a generalized linear modelwith a quasibinomial reference distribution. We applied a Tobit regression to the time spent in the refuges because the distribution of the variable was similar to a censored Normal distribution [95]. Number of Headbobs and first access to ZS were analysed using a generalized linear model with a negative binomial distribution as link function to avoid overdispersion. All analyses were performed in the R environment [96]. We calculated the following indexes of effect size: the R-squared for the ANOVAs, and Mcfadden Index and the pseudo R-squared for logistic and Tobit regressions, respectively [97]. For the Kruskal-Wallis tests, we calculated the Eta squared [98].

### 2.4 Ethical statements

Permission for field behavioural recordings, scientific capture and experimentation was granted by the provincial government environment agency (Secretaría de Ambiente y Cambio Climático–permit number: 546833053717) and was approved by the IDEA animal welfare committee (protocol numbers: 02/2017 and 12/2019).

## 3. Results

### 3.1. Field study: Activity pattern and social interactions

#### 3.1.1 Characterization of thermal habitats

**Fig 2:**
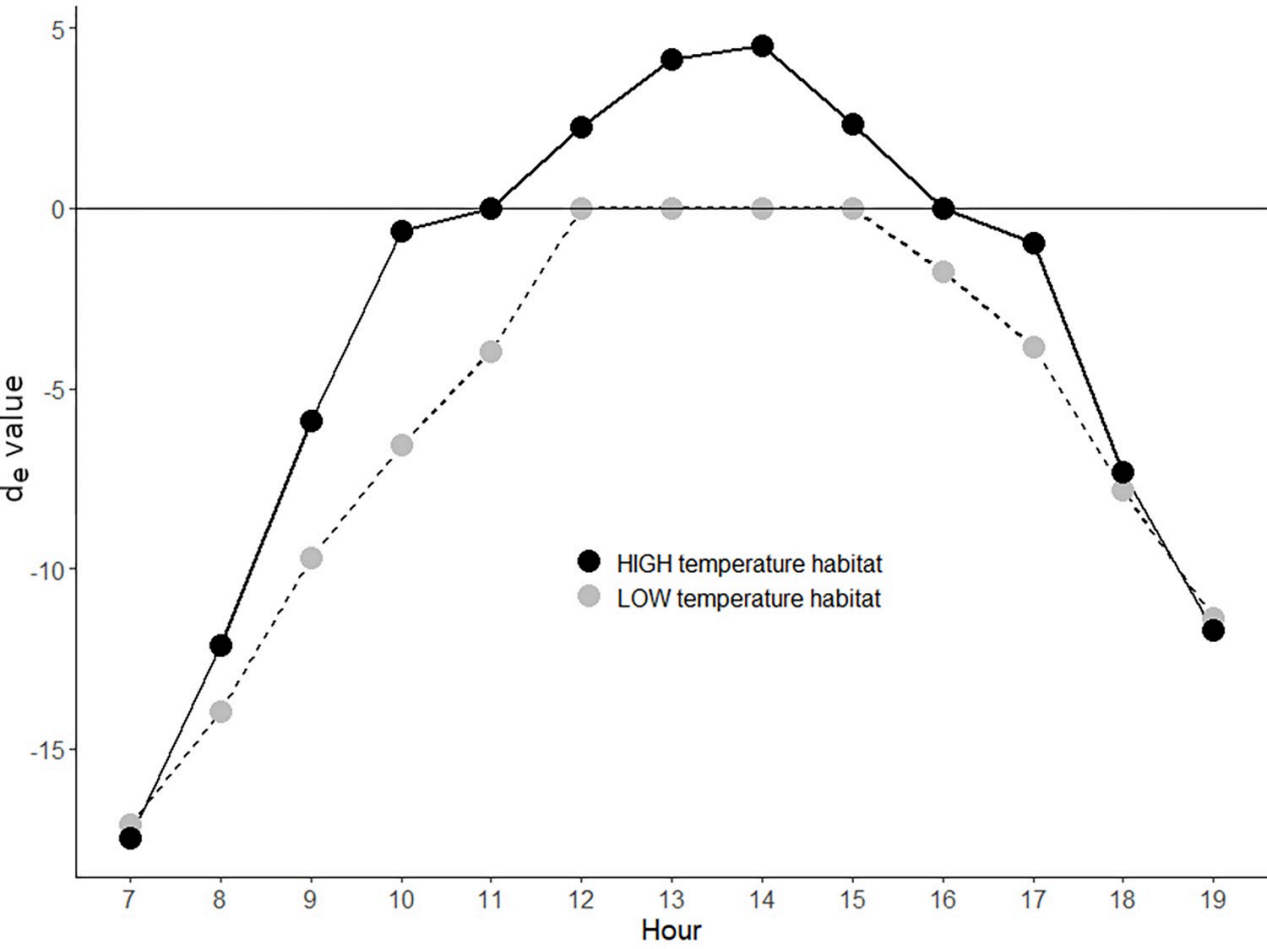
Characterization of thermal habitats used by *Tropidurus spinulosus* according to the *d*_*e*_ index.

The HIGH and LOW habitats were thermally different (HIGH: Ts = 30.23 ± 9.82°C and LOW Ts = 26.60 **±** 8.39°C). In the HIGH habitat, Ts was within the Tset range of the species between 1100–1200 hours and between 1600–1700 hours. Outside these two segments, the *d*_*e*_ index abruptly departed from zero, especially during the central hours of the day, when substrate temperatures peaked. In the LOW habitat, Ts was within the Tset range at 1300–1400 hours and the *d*_*e*_ index varied more gradually around the peak, indicating slower heating and cooling of the substrate (Fig 2; Table 1).

**Table 1:** P-values of the hourly *d*_*e*_ index.

Hour	*d*_*e*_ Index HIGH	*d*_*e*_ Index LOW	p-val HIGH-LOW	p-val-HIGH-Zero	p-val-LOW-Zero
7	-17.45	-17.07	0.45	<0.01	<0.01
8	-12.13	-13.93	<0.01	<0.01	<0.01
9	-6.17	-9.81	<0.01	<0.01	<0.01
10	-1.70	-6.93	<0.01	<0.01	<0.01
11	-0.90	-4.62	<0.01	0.2	<0.01
12	2.80	-0.84	<0.01	<0.01	0.13
13	4.48	0.03	<0.01	<0.01	0.94
14	4.81	-0.61	<0.01	<0.01	0.28
15	3.08	-1.30	<0.01	<0.01	0.01
16	0.92	-2.70	<0.01	0.3	<0.01
17	-2.10	-4.43	<0.01	<0.01	<0.01
18	-7.45	-7.88	0.13	<0.01	<0.01
19	-11.69	-11.36	0.49	<0.01	<0.01

T-tests were applied to evaluate differences between habitats (p-val HIGH-LOW) and between each habitat and the set-point range of species (*d*_*e*_ index = 0; p-val-HIGH-Zero and p-val-LOW-Zero).

#### 3.1.2. Daily patterns

Lizards were generally active throughout the recorded hours in both HIGH and LOW habitats, with almost no activity being recorded before 0800 hours and after 1800 hours (Table 2).

**Table 2:** Comparison of activity pattern and social interactions between HIGH and LOW thermal habitats along the hourly segments.

Hour	Activity			Simultaneous presence			Pushups			Movement		
	High	Low	P-value	High	Low	P-value	High	Low	P-value	High	Low	P-value
7–8	60.6	0.0	<0.01	NA			NA			NA		
8–9	1398.0	578.8	<0.01	507.4	0.0	0.01	67.4	44.8	0.03	88.4	18.8	<0.01
9–10	1775.4	287.2	<0.01	279.0	60.8	<0.01	69.6	4.6	<0.01	71.0	25.0	<0.01
10–11	2287.8	1712.4	<0.01	463.6	74.4	<0.01	107.6	86.2	0.12	102.2	104	0.9
11–12	2344.6	1300.6	<0.01	846.0	57.8	<0.01	83.6	50.8	<0.01	70.2	68.2	0.8
12–13	1209.2	3478.6	<0.01	339.8	317.8	0.3	121.8	104.8	0.25	97.2	157.4	<0.01
13–14	511.4	2513.2	<0.01	78.0	99.2	0.1	70.4	98.2	0.03	54.4	112.6	<0.01
14–15	489.2	3410	<0.01	378.2	285.8	<0.01	75.8	73.8	0.8	96.4	232.6	<0.01
15–16	619.8	1940.8	<0.01	121.4	161.6	0.02	36.2	43	0.4	60.0	81.4	0.07
16–17	1707.6	1658	0.4	588.6	16.8	<0.01	67.2	37.4	<0.01	119.4	37	<0.01
17–18	2207.0	1032	<0.01	558.8	62.6	<0.01	36.8	23.6	0.08	66.6	26.6	<0.01
18–19	674.4	719.4	0.65	178.8	0	<0.01	8.4	25	<0.01	20.6	14.4	0.3

Variables are expressed in seconds. P-values of the Chi-square test are reported. Significant differences are highlighted in dark grey, indicating that behavioural activity was higher in the HIGH than in the LOW temperature habitat, and in light grey, indicating the opposite pattern. NA: not applicable (missing data)

**Fig 3:**
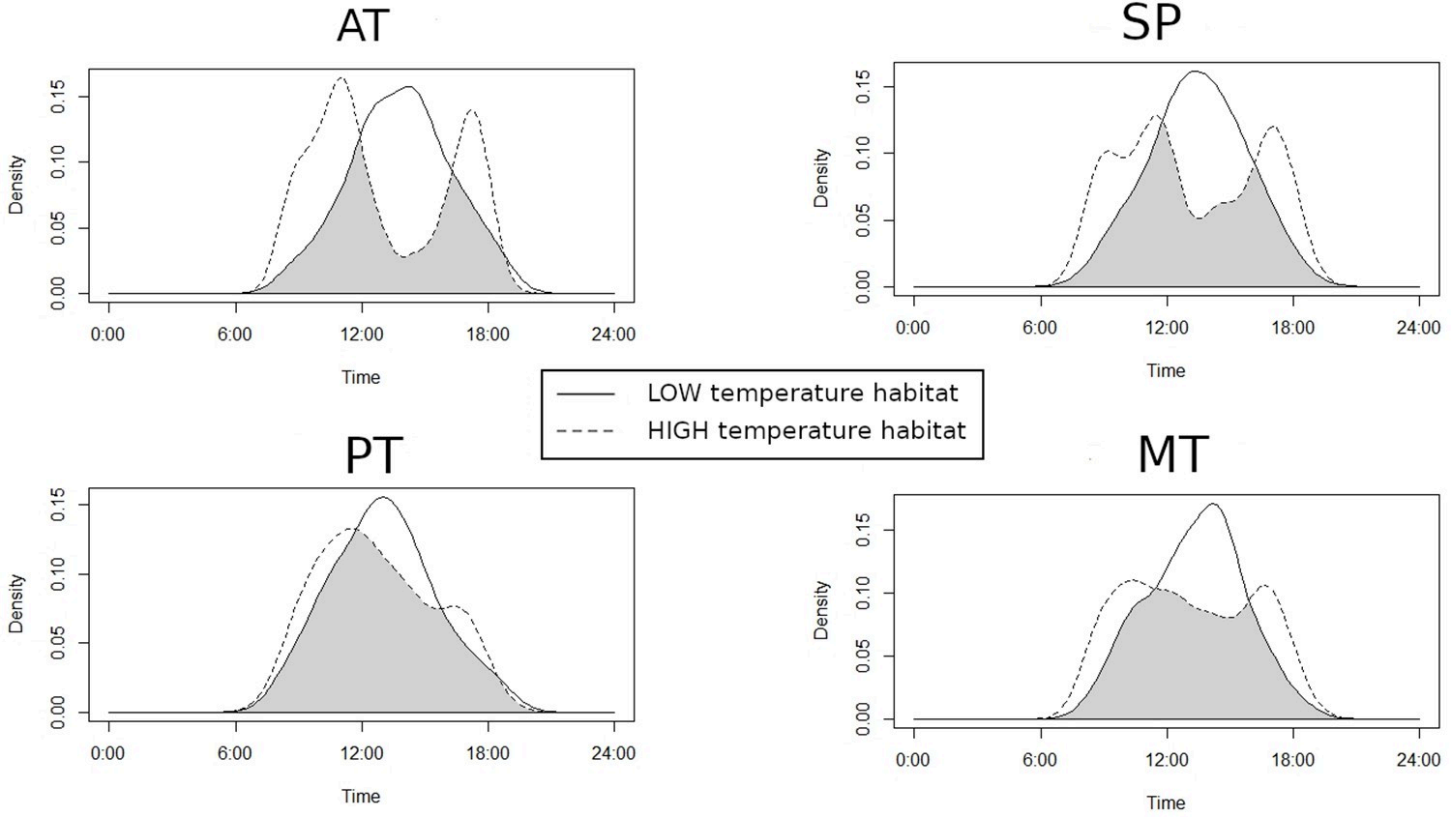
Patterns of activity and behaviour of *Tropidurus spinulosus* in HIGH and LOW temperature habitats. AT = Activity Time, SP = Simultaneous Presence, PT = Pushups Time, MT = Movement Time.

In the HIGH habitat, Activity Time (AT) showed a bimodal pattern, with peaks at 1100 hours and 1500 hours, whereas in the LOW habitat, the pattern was unimodal since activity was concentrated at around 1200 and 1300 hours (Fig 3). The time that individuals were active (AT) in each hourly segment differed between thermal habitats, being higher in the LOW habitat during the central hours of the day (Table 2). Nevertheless, lizards from both habitats remained active for the same amount of time during the day (Table 3).

**Table 3:** Overlap and total time of activity pattern variables in *Tropidurus spinulosus* in HIGH and LOW thermal habitats.

Variable	Overlap Coefficient	Bootstrap mean	Confidence interval	P-value	Daily Total time HIGH	Daily Total time LOW	P-value
AT	0.59	0.64	0.52–0.66	<0.01	96.29 ± 389.73	90.61 ± 342.28	Z = -1.08, p = 0.30
MT	0.76	0.79	0.68–0.84	0.30	3.42 ± 12.52	3.83 ± 13.53	Z = -0.86, p = 0.38
SP	0.68	0.72	0.47–0.80	0.60	59.14±213.72	11.16±64.31	Z = -2.37, p = 0.05
PT	0.86	0.87	0.78–0.95	0.36	3.01 ± 12.25	2.58± 10.77	Z = -0.65, P = 0.51

Overlap coefficient bootstrap and its associated confidence interval are reported; for total activity time, Z and P-values of the ANOVA are reported. AT = Activity Time, MT = Movement Time, SP = Simultaneous Presence, PT = Pushups Time.

Movement Time (MT) showed a sharp unimodal pattern in the LOW habitat, always around 1200–1300 hours, whereas in the HIGH habitat lizards displaced in a uniform pattern from 1100 to 1500 hours, with no observable peak (Fig 3). The time that lizards spent moving on the rock surface significantly varied between habitats at different times of the day. Early in the morning (0800–1000 hours) and at mid-afternoon (1600–1800 hours) lizards moved in the HIGH habitat for a longer time, whereas during the central hours (1200–1600 hours) lizards moved in the LOW habitat for a longer time (Table 2). MT overlap between habitats was high, and there was no difference in total daily MT (Table 3).

Simultaneous Presence (SP) roughly followed AT, with two peaks at 1100 and 1500 hours in the HIGH habitat, and a peak at 1100–1300 hours in the LOW habitat (Fig 3C). However, the difference between the two daily patterns was not significant. The total daily time of SP was higher in the HIGH habitat and accordingly the hourly SP varied in most time intervals during the day (Tables 2, 3).

Pushups Time (PT) was unimodal in both habitats, with a peak from 1000 to 1200 hours (Fig 3D) and a high overlap coefficient, with no difference in the total time allocated to this display between habitats (Table 3). Regarding the hourly segments, the time that lizards spent performing Pushups was similar between habitats in almost all segments; however, sometimes it was higher in the HIGH habitat (Table 2).

### 3.2 Laboratory experimental study: Mate choice

**Fig 4:**
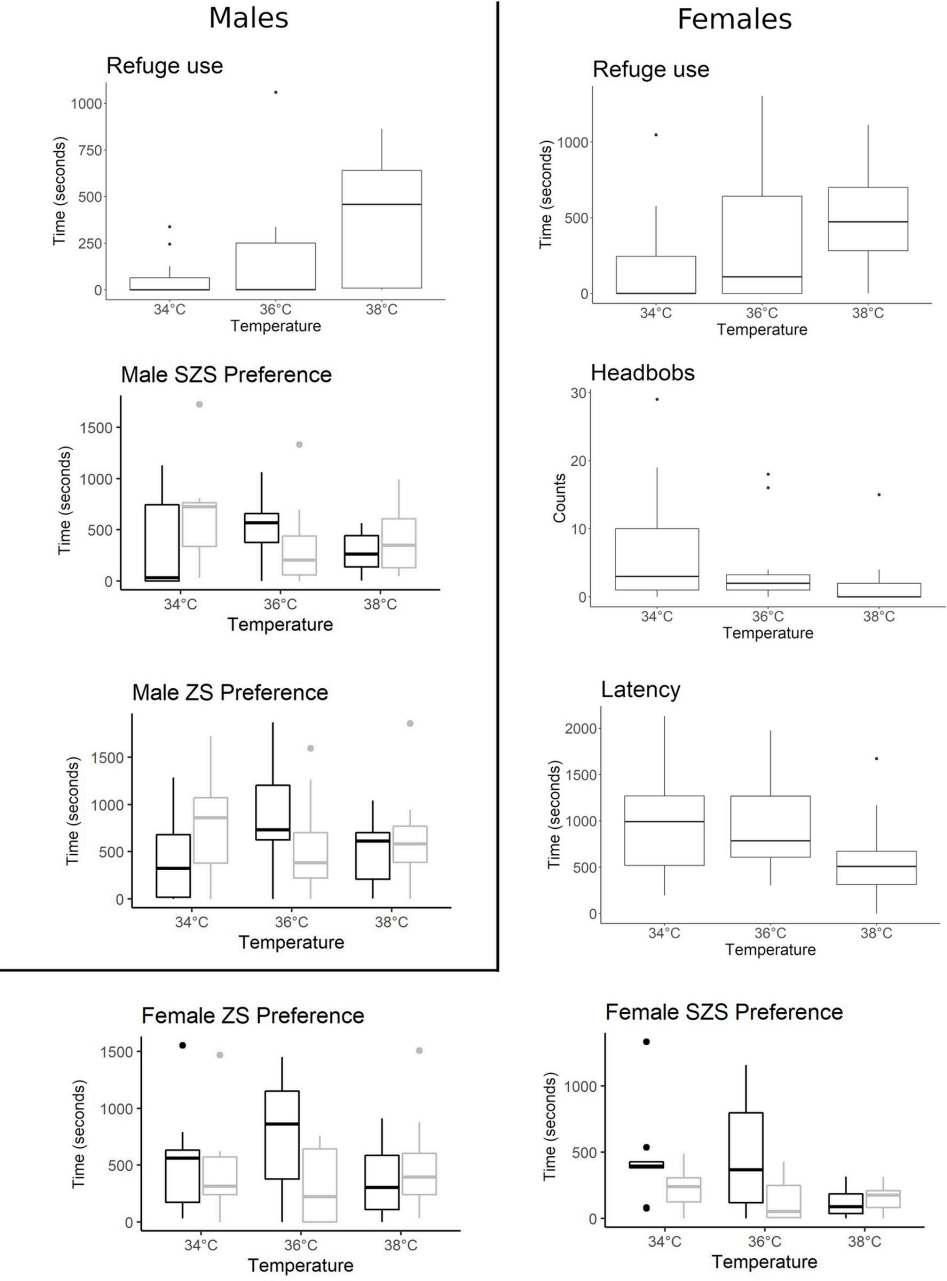
Behavioural variation during mate choice trials in relation to thermal treatments in *Tropidurus spinulosus*. For female preference, black and grey boxplots highlight the time that the focal female spent with the colourful and dull male respectively (colour sum). For male preference, black and grey boxplots indicate the time that the focal male spent with the large and small female, respectively.

In general, males had to increase the use of refuges at high temperatures (Tukey 34°C vs 38°C, P = 0.01; Table 4, Fig 4), although with a high inter-individual variability (Male refuge use variance: 34°C = 11269, 36°C = 78654, 38°C = 107728), whereas females showed the same tendency but with only marginal significance (Tukey 34°C vs 38°C P = 0.07). Moreover, females showed a tendency for a shorter latency time at high temperatures (Tukey 34°C vs 38°, P = 0.09; Table 4, Fig 4). No effects of thermal treatments were found for Movement, First Access or NRZ for either males or females.

**Table 4:** Effect of temperature treatments (34°C, 36°C and 38°C) on mate choice for *Tropidurus spinulosus* males and females.

Variable	Males					
	Mean 34°C	Mean 36°C	Mean 38°C	Statistics	P-value	Effect size
**Refuge (Time)**	60.57 ± 106.15	162.38± 280.45	386.01 ± 328.22	Dev = 10.46	**P<0.01**	Mcf = 0.02
**Time preference SZS “Strict” (time proportion spent with the Larger female)**	0.27±0.28	0.61±0.33	0.47±0.33	Chisq = 4.90	P = 0.08	Odds = -1.05
**Time preference ZS “Broad” (time proportion spent with the Larger female)**	0.34±0.24	0.58±0.32	0.43±0.27	Chisq = 6.63	**P = 0.05**	Odds = 1.83
**Pushups (Time)**	22.26± 22.95	24.30± 21.28	21.51± 17.98	F = 0.01	P = 0.98	R^2^ = 0.03
**Movement (Time)**	14596.54± 10815.63	14918.28± 13716.20	12039.36± 11429.71	F = 1.27	P = 0.29	R^2^ = 0.01
**Latency (Time)**	983.5± 520.60	817.33± 661.88	823.43± 495.18	F = 0.39	P = 0.67	R^2^ = 0.02
**Headbobs (Counts)**	5.50± 15.30	8.27± 17.13	11.59± 27.47	Dev = 2.12	P = 0.34	Mcf = 0.01. pR^2^ = 0.04
**First Access (Counts)**	Larger = 5 Smaller = 9	Larger = 10 Smaller = 4 No Pref = 1	Larger = 7 Smaller = 7	LR-Chisq = 3.72	P = 0.15	Mcf = 0.06. pR^2^ = 0.11
**NRZ (Time)**	1398.17±875.92	1283.44±803.51	1487.36±535.51	Chisq = 0.26	P = 0.76	pR^2^ = 0.02
**Variable**	**Females**					
**Refuge (Time)**	197.58± 323.16	368.85± 458.40	496.96± 360.70	Dev = 4.84	P = 0.08	Mcf = 0.01
**Preference SZS “Strict” (time proportion spent with the Colourful male)**	0.60±0.20	0.66±0.37	0.45±0.36	Colour sum: Chisq = 6.37	**P = 0.04**	Odds = 1.46
	0.60±0.20	0.66±0.37	0.53±0.36	Yellow: Chisq = 4.39	P = 0.11	Odds = 1.21
**Preference ZS “Broad” (time proportion spent with the Colourful male)**	0.51±0.24	0.64±0.35	0.47±0.27	Colour sum: Chisq = 4.52	**P = 0.03**	Odds = 1.05
	0.51±0.24	0.64±0.35	0.48±0.27	Yellow: Chisq = 4.21	**P = 0.04**	Odds = 1.01
**Pushups (Time)**	-	-	-	-	-	-
**Movement (Time)**	9328.267± 7682.54	14138.403±13677.83	10873.442± 12636.41	F = 1.53	P = 0.46	R^2^ = 0.01
**Latency (Time)**	1026.16± 616.88	973.25± 599.53	569.84± 458.60	F = 2.51	P = 0.09	R^2^ = 0.10
**Headbobs (Counts)**	3.31± 8.91	1.93± 6.05	1.04± 4.14	Dev = 4.66	P = 0.09	Mcf = 0.02. pR^2^ = 0.12
**First Access (Counts)**	Colourful = 4 Not Colourful = 7 No pref = 1	Colourful = 8 Not colourful = 4	Colourful = 7 Not colourful = 5 No pref = 1	LR-Chisq = 2.26	P = 0.32	Mcf = 0.04. pR^2^ = 0.08
**NRZ (Time)**	1463.39± 494.34	1338.06± 601.42	1635.100± 850.58	Chisq = 1.72	P = 0.42	pR^2^ = 0.01

The following test statistics are reported: Log-likelihoods for Tobit regression (“Log-lik”); F statistics for ANOVAs; deviance values for the negative binomial (“Dev”); LR-Chisq for the Chi square test for the logistic regressions. “Strict” preference refers to preference calculated according to SZSs, and “broad” preference refers to ZSs. NRZ: near the refuge zone. In First Access, “Larger” stands for the number of times the individual accessed the selection zone of the larger female of the dyad as the first option, whereas “Smaller” refers to the smaller female of the dyad and “No Pref” indicates that the individual did not access any ZS during the trial. First Access “Colourful” indicates the number of times the focal female accessed first the ZS of the male with the highest colour sum/yellow extension, whereas “Not colourful” refers to the male with the lowest colour sum/yellow extension and “No pref” indicates that the female did not access any ZS during the trial. Significant p-values are highlighted in bold.

According to SZS, females showed a tendency to prefer colourful males (colour sum) at 34°C (0.6 probability; Intercept P = 0.06), which increased at 36°C (0.66 probability) and diminished significantly at 38°C (0.45 probability; Tukey 36°C vs 38°C, P = 0.03; Table 4, Fig 4), this pattern is similar for ZS. However, the results were less clear-cut when yellow is considered as colour stimulus since at 38°C the preference for yellow males decrease less sharply (0.53 probability). Males showed a tendency to select small females at 34°C for SZS (0.27 probability of selecting large females, Intercept P = 0.09), whereas at 36°C males showed a marginally significant preference for large females (0.61 probability, Tukey 34°C vs 36°C, P = 0.08; Table 4). For ZS broad preference, males showed the same pattern with higher significance (Tukey 34°C vs 36°C, P = 0.02; Table 4; Fig 4).

Regarding behavioural displays, male Pushups were not affected by the temperature treatment. Headbobs showed a marginally significant result for females, who tended to perform fewer displays at increasing temperatures (Tukey 34°C vs 38°C, P = 0.08; Table 4). In males, the variability in Headbobs displays rose at increasing temperatures (Headbobs variance: 34°C = 234; 36°C = 293; 38°C = 755).

## 4. Discussion

As we predicted, the behavioural activity pattern of *Tropidurus spinulosus* in its natural habitat varied between two different thermal conditions. Likewise, lizards from the two thermal habitats differed in the hourly adjustment of time devoted to movement and time spent in the presence of conspecifics. However, the total active time, total movement time and the temporal pattern of Pushups were similar in both habitats. Therefore, our predictions were partially corroborated, since the hourly and daily social behavioural pattern varied (being unimodal in the LOW temperature habitat and bimodal in the HIGH temperature habitat), but the total daily activity time did not. Considering sexual interactions, as we predicted, males allocated less time to interact with conspecifics in the highest temperature treatment than in the lowest one, because they spent more time inside the thermal refuges; females showed the same pattern, but it was marginally significant. High temperatures also modified the selection outcome and the Headbobs pattern for males and females. Thus, in the present study, the hypothesis that postulates a reduction in time allocated to mate choice and modification of the selection outcome caused by temperature increments was corroborated in lizards for the first time.

Increasing temperatures are likely to reduce the width of the operational thermal window in lizards. However, understanding how lizards react to changing temperatures requires the study of the pattern of behavioural changes [8]. Although we found no differences in total activity time between habitats, the behavioural activity pattern varied, since the overlap between HIGH and LOW temperature habitats was low according to the criteria proposed by [99]. The transition between a unimodal and a bimodal pattern shows that lizards modify their behaviour in a plastic manner along a temporal axis, possibly because other behavioural options, such as posture modification, have already been depleted [100, 101]. Similarly, species in wide altitudinal gradients rely on behavioural thermoregulation, modifying hourly activity [38, 102]. *T*. *spinulosus* showed low activity levels when operative temperatures were outside the species’ preferred range, which coincides with the pattern found in other ectotherms [103, 104]. This finding suggests that high temperatures may constrain activity, as predicted by “medium constraint” theoretical models, which postulate restricted activity within the Tset boundaries [8, 105, 106]. Long-term effects of these restrictions may force a species to choose one activity over another [4]. However, the activity pattern we recorded is just one dimension of activity, and to get a more comprehensive picture of the species plasticity, further research should address other relevant aspects of activity in relation to temperature, such as probability, mode and vigour/physical exertion [16].

The total Movement time was the same in the two habitats; however, the hourly pattern varied. Movement was unimodal in the LOW temperature habitat, whereas in the HIGH temperature habitat it was approximately uniform throughout the day. The pattern we observed for this variable may highlight another aspect of plasticity, the “mode”, which corresponds to the different types of activities in which lizards can engage outside the refuges (movements, courtship, social displays, etc.) and each type may show a different relationship with temperature [6, 16]. Some species and/or populations show a positive relationship of home range size and the frequency of high-displacement movements with the time spent by individuals within their optimal temperature range [6]. Likewise, other ectotherm species generally concentrate locomotor activity when operative temperatures are within the preferred range [107], as observed in the movement pattern of the LOW temperature habitat. However, under warmer conditions lizards may also perform locomotor activity across a wider range of temperatures, which explains the uniform pattern of the HIGH temperature habitat. This plasticity has also been indicated for other species, such as *Norops cristatellus*, in which peak activity occurs approximately at the preferred body temperature, although locomotion and sprints can occur even at higher temperatures [16]. Yet, in lizards, performing an activity such as locomotion under thermal stress may trigger thermal stress responses and impose new energetic costs, which may induce individuals to allocate less energy to other activities/traits [108].

Lizards tend to aggregate when operative temperatures are close to their preferred range, since many social activities are energetically demanding [16, 101, 109]. Activity results showed that lizards from both habitats tended to aggregate mainly when Te was within Tset. However, lizards in the HIGH temperature habitat were observed in groups for longer periods than those in the LOW temperature habitat, and maintained social interaction even outside the Tset range. The capacity of movement observed at high Te in the HIGH temperature habitat may also have favoured aggregation, possibly enabling intra- and inter-sexual encounters [109]. Therefore, our hypothesis of “missed opportunities” for social interactions is not supported for the lizards of the HIGH habitat. However, the fact that sometimes they have to aggregate at unsuitable temperatures may modify precopulatory sexual selection dynamics, such as mate choice (see below). Furthermore, our results reinforce the hypothesis of a variable relationship between different behaviours and the thermal environment [6, 8, 16]. Accordingly, Pushups showed the highest overlap between habitats at the central hours of the day. Even in the HIGH habitat, Pushups were performed when substrate temperatures were not suitable; this activity possibly entailed metabolic risks since under such conditions energetic costs are probably high [62, 110]. Similarly, some species of the genus *Norops* concentrate the production of visual displays at the same times of the day, even though those species are under different thermal ranges [111]. Nevertheless, performing displays outside the preferred temperature range may lead to energetic costs that hinder further displays throughout the day. Indeed, in our study *T*. *spinulosus* individuals in the HIGH habitat did not perform Pushups during the afternoon, when there was simultaneous presence of conspecifics. The lack of connection between behavioural displays and levels of social activity was also observed in *Norops* species [111]. Therefore, it is important to underline our space-for-time study design used to elucidate social interaction shows that high temperatures could lead to the decoupling of thermal environment and social aggregation from behavioural communication.

Mate choice dynamics was also affected by substrate temperature through the reduction of the time lizards allocated to interactions with the possible partners and the modification of the patterns of selection. Indeed, this is the first study that tested in lizards the theoretical hypothesis of “missed opportunities”, which postulates a reduction in time allocated to mate choice in relation with increased temperature [4]. *T*. *spinulosus* males had to increase the use of refuges at high temperatures, reducing 3 to 4 times the time they interact with possible partners. However, for females this pattern was less marked. The increased use of refuges indicates that *T*. *spinulosus* was not able to withstand temperatures in the upper set point range for a long time, which is consistent with the results of the daily activity pattern. Interestingly, in the mate choice trials, the moderate increment in refuge use and the shorter latency shown at high temperatures by females while keeping sexual interactions may be associated with females’ narrow reproductive window in some lizards [112, 113]. In contrast, the marked increase in the use of refuges by males may be related to the protection of their sperm, since its performance may be hindered by high temperatures [114]. Moreover, the need to seek refuge from high temperatures and increase the hours of restriction could leave less room for courtship dynamics and may force individuals to choose their partners hastily, thus modifying the pattern of selection.

Females chose the male with the largest colour area at the preferred temperature of the species and they reinforced this choice at intermediate temperatures, whereas at high temperatures they showed a more random pattern. However, if we consider only the yellow band of the flanks as the male trait tested for SZS, the pattern of selection is similar although less marked. Although in our dataset the yellow band was the best predictor of female mate choice, the highest stimulation of the lizard vision is caused by the three colour bands together (yellow, green and blue) [49]. Moreover, blue colouration is related to important reproductive population parameters, since a high extension of this band can be found when the operative sex ratio is strongly biased towards males [72] and could therefore be relevant in a mate choice context. In other species as well, the contrast between different colour hues can provide a higher stimulation to the lizard eye, for example the contrast between UV-blue scales and the red ventral colouration in Lacertidae [115]. Male colouration has often been related to traits linked to fitness like body size, reproductive tactics and immune response [116–118]; therefore female mate choice based on colouration as an honest signal may benefit the reproductive process. However, our results show that at high temperatures, colour bands together could not be selected by females due to a modification of the female selection process, since their attention may be focused on managing heat stress [40], ultimately leading to female tendency to select males more randomly. For females, the temperature-driven modification of mate choice outcome coupled with a marginal increase in refuge use and the lower latency at high temperatures may reinforce the idea of a “rushed mate choice”. Thus females would make little distinction between males with different phenotypes. Moreover, it would be interesting to analyse in more detail how male colouration changes at increasing temperatures, since the first evidence in our study species suggests that high temperatures affects males’ plastic capacity of darkening the ventral colouration [50].

On the other hand, the males’ pattern shows that a moderate increment in temperature could cause a shift in mate choice, even though the pattern is not clear-cut: at the preferred temperature of the species (34°C) there was a tendency of selection towards small females, whereas, at intermediate temperatures (36°C) evidence of selection towards larger females was weak. This result is particularly interesting since in the Squamata, males usually tend to prefer larger females due to their higher fecundity [60]. However, the tendency to select small females could be influenced by other relevant traits in male selection, such as the female body condition. This hypothesis should be tested, since it could play a role in signalling reproductive potential of females. For example, in *Salvator merianae* the reproductive males are more abundant when there is a high frequency of medium-large females with high fat storage [60]. Further research is needed to understand the potential change in male selection of female body size.

Behavioural displays were differentially affected by temperature between sexes, since females tended to decrease the production of Headbobs at high temperatures (Fig 4), whereas males incremented Headbobs variance. The reduction of Headbobs displays in females can be coupled to the increasing random tendency in mate preference, suggesting that they invest less energy in interacting with the dyad individuals to evaluate them at high temperatures than at lower temperatures. The increase in male variability in Headbobs production is interesting, since it may highlight an individual’s specific motivation to perform behavioural displays in different environmental contexts [119]. In some lizard species, Headbobs are slightly more effective than Pushups in reducing the amount of female rejection, however their combined effect always elicits a stronger positive response [66]. Despite the cost of Pushups [61], in our trials, males did not reduce the frequency of this behaviour; instead, some males incremented Headbobs at intermediate and extreme temperatures. This suggests that, with an increase in temperature, some males may try to “intensify” the courtship behaviours by relying on Headbobs, which may be key in reducing female rejection. Other males may have not performed further behavioural displays at high temperature to avoid overheating, since heat stress responses may increase the energetic costs of movement and behaviour [5]. The high inter-individual variation in the performance of behavioural displays as well as the time spent choosing a female at high temperatures may be related to different individual reproductive strategies or to a high variability in thermal tolerance between males [120].

In conclusion, increasing temperatures may modify the behavioural activity pattern, since lizards shift from a unimodal to bimodal daily pattern. Social behaviour could also be impacted, since individuals adjust interactions to operative temperatures close to their preferred range. However, lizards might sometimes move between perch positions and interact even outside this range. On the other hand, behavioural displays like Pushups would be less plastic in a warming context, possible leading to a decoupling of social aggregation from behavioural communication. Furthermore, high temperatures also affect mate choice dynamics, since both males and females spent less time interacting with possible partners, thus missing opportunities. Moreover, the selection outcome may become increasingly random, leading to different reproductive strategies to cope with thermal stress. Therefore, increasing temperatures may trigger plastic adjustments that will affect the behavioural activity pattern and intersexual interactions. The consequences of such adjustments should be tackled in the future, since they may affect individual fitness and, consequently, population dynamics and evolution of reproductive traits.
